# The increasing variability of tropical cyclone lifetime maximum intensity

**DOI:** 10.1038/s41598-018-35131-x

**Published:** 2018-11-09

**Authors:** Jinjie Song, Philip J. Klotzbach, Jianping Tang, Yuan Wang

**Affiliations:** 1 Key Laboratory of Mesoscale Severe Weather, Ministry of Education, Nanjing, China; 2 School of Atmospheric Sciences, Nanjing University, Nanjing, China; 3 Joint Center for Atmospheric Radar Research of CMA/NJU, Nanjing, China; 4 Department of Atmospheric Science, Colorado State University, Fort Collins, USA

## Abstract

This study investigates long-term changes in the variability of TC intensity of global tropical cyclones, a topic which has been relatively infrequently studied to date. Our study finds that the variability of global TC lifetime maximum intensity (LMI), as measured by the LMI standard deviation, increases during 1981–2016. The increasing trend in LMI variability is statistically significant for both the Northern and Southern Hemispheres, with three individual TC basins: the western North Pacific, the South Indian and the South Pacific also having statistically significant increases. This increasing trend primarily results from distinct changes in the relative percentages of TCs with different intensities. When comparing two periods: 1981–1998 and 1999–2016, the proportions of weak and strong TCs increase, whereas moderate TCs occur relatively less frequently. This bimodal pattern of observed LMI distribution change is further linked to opposite trends in the average intensities of TCs that undergo rapid intensification (RI) during their lifetime (RI TCs) and those that do not (non-RI TCs). The LMI distributions of RI and non-RI TCs migrate to higher and lower intensities, respectively. Our results demonstrate from an observational perspective that strong TCs have strengthened while weak TCs have weakened as the global climate has warmed since 1981.

## Introduction

The influence of climate change on tropical cyclone (TC) activity has generated considerable scientific attention during the past ~20 years^1–3^. A majority of modelling studies estimate a decrease in the total frequency of global TCs accompanied by an increase in the average number of intense TCs in response to anthropogenic global warming^2,4,5^. This anticipates a future increasing tendency in the average intensity of global TCs, which is consistent with significant increasing trends in both the mean lifetime maximum intensity (LMI) of TCs and the intensity of the strongest TCs derived from historical satellite-based temporally homogenized datasets^6–11]^. By contrast, a few publications project increasing TC frequency^12,13^. Due to the lack of theoretical understanding of storm occurrence^3,8^, there is greater inconsistency in projections of future TC frequency than intensity. It also remains uncertain how global warming affects changes in LMI variability for global TCs as well as individual basin TCs. This uncertainty can influence both the verification of TC simulations in climate models and the prediction of TC activities in different intensity categories.

LMI variability is primarily determined by the probability density function (PDF) of LMI, serving as a fundamental feature of the TC climatology^7,14^. There exists a bimodal pattern of storm LMI PDFs over the globe as well as for individual TC basins^7,13,15–17^. Two peaks in the PDF are separately related to LMI distributions of TCs that undergo rapid intensification (RI) during their lifetime (RI TCs) and TCs that do not (non-RI TCs)^17^. In several modeling studies, both the overall LMI distribution and the peak of the LMI PDF are skewed to higher intensities in a warmer climate, which induces an increasing and decreasing proportion of strong and weak TCs, respectively^18–26^. This change could significantly modulate the LMI mean but also influence the LMI variance. In contrast, a few climate models represent a more complex change in the LMI PDF under high-CO_2_ conditions^27,28^. Although the majority of the LMI PDFs are shifted to higher intensities in warmer scenarios, the relative frequency of weak TCs also increased in some simulations^27,28^. However, this feature was rarely discussed in the literature, perhaps because there exists a great deal of disagreement as to the definition of weak TCs in climate model outputs. Since TCs in climate models are usually defined by objective tracking algorithms, the identified storm activity is highly sensitive to the specific thresholds in tracking techniques from different modeling studies which utilize different models and may have different model resolutions^29,30^. Compared with strong TCs, it is harder to correctly identify weak TCs using various tracking methods in climate models, which results from the challenges involved in separating weak TCs from disturbances and waves. Due to these uncertainties, modeled activity of weak TCs is not discussed as extensively as that of strong TCs in most previous studies. Moreover, compared with modeling studies, there have been few publications on the observed changes of the PDF of LMI. Many modeling papers utilize the observed LMI PDF to verify their historical simulations and then project future changes in the simulated LMI PDF (e.g. ref.^13^).

In this study, we utilize TC LMI records from the National Hurricane Center (NHC)^31^ and the Joint Typhoon Warning Center (JTWC)^32^ as archived in the International Best Track Archive for Climate Stewardship (IBTrACS)^33^ v03r10. We use data during the satellite era (1981–2016) to investigate the potential long-term trend in TC LMI variability, which is measured by the standard deviation (STD) of the annual LMI sample. We find that there exist significant increasing trends in the annual TC LMI STD over the globe as well as for some individual basins. These trends are primarily caused by differences in the LMI PDF between the two sub-periods (1999–2016 minus 1981–1998), which represents a bimodal pattern with two peaks occurring at higher and lower intensities. Furthermore, the different trends in annual averaged intensities of RI TCs and non-RI TCs are responsible for the bimodality of the distribution of the LMI PDF change. Here RI is defined as an increase of at least 30 kt in TC intensity within a period of 24 h or less^34,35^.

## Results

### Increasing variability of TC LMI

**Figure 1:**
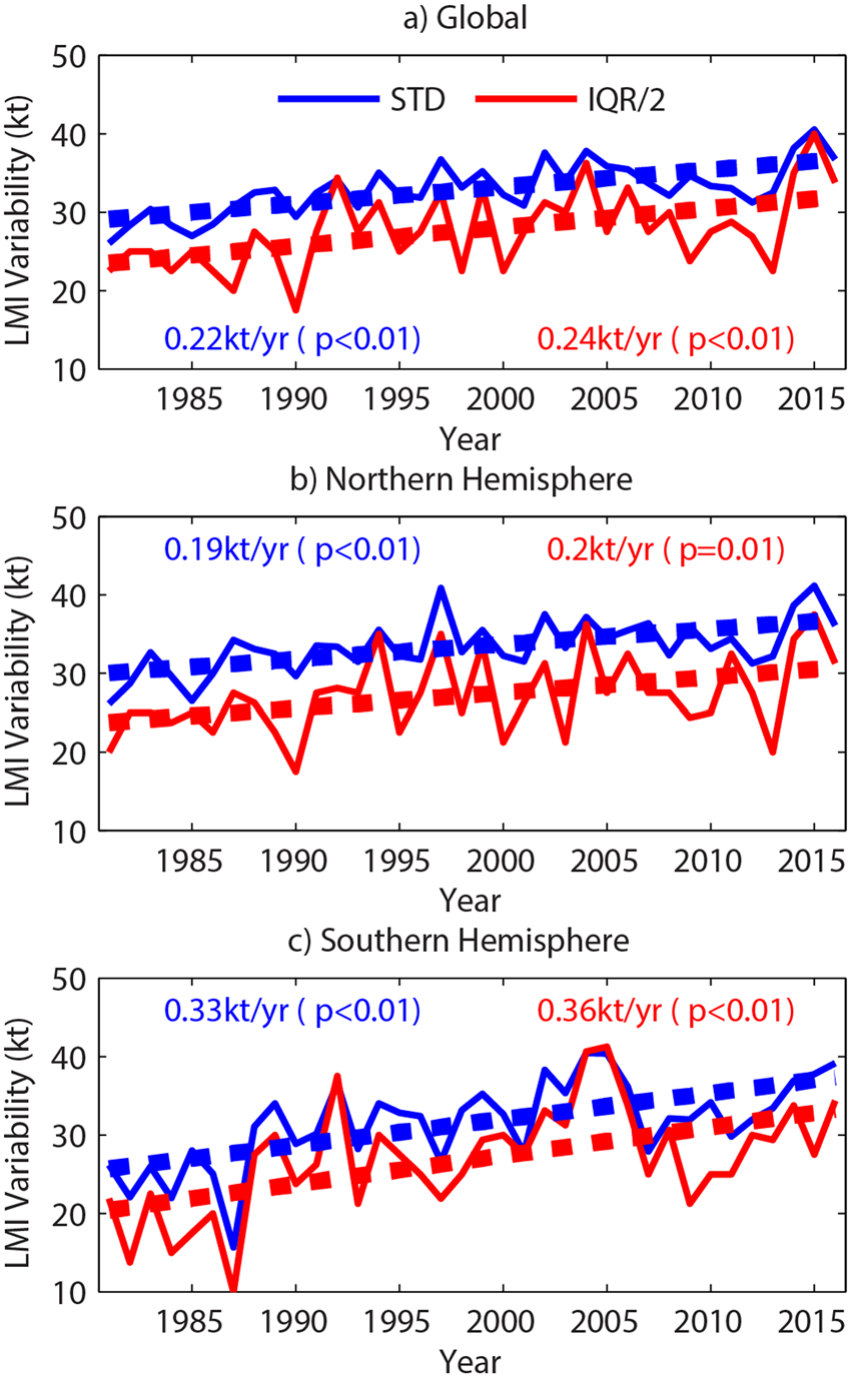
Increasing variability in TC LMI. Time series of annual STD and half IQR of TC LMI from 1981 to 2016 and associated linear trend lines over the globe (**a**), the Northern Hemisphere (**b**) and the Southern Hemisphere (**c**). Blue and red lines refer to the STD and half IQR, respectively. The slope of the trend line and its significance level are shown in the plots.

When the annual STD of LMI is computed from the IBTrACS data during 1981–2016, a significant upward trend is represented over the globe, with an increasing rate of 0.22 kt yr^−1^ (Fig. 1a). There are also significant increasing trends in the annual LMI STDs over both hemispheres (Fig. 1b,c), with the rate in the Southern Hemisphere (SH) being much larger than that in the Northern Hemisphere (NH). All individual ocean basins make positive contributions to the hemispheric trends (Extended Fig. 1), which indicates that the increasing LMI STD is a global phenomenon. However, there are considerable differences in both the trend amplitudes and statistical significance levels for individual TC basins. The increasing trend in LMI STD is most significant over the western North Pacific (WP). Since this is the basin with the most storm occurrences, the WP is the greatest contributor to the NH trend in LMI STD. Although the increasing LMI STD rate over the North Indian Ocean (NI) is greater than that over the WP, it is not statistically significant due to the large variability in interannual variations of the LMI STD. By comparison, there are only slightly increasing trends in LMI STD over the North Atlantic (NA) and the eastern North Pacific (EP). The South Indian Ocean (SI) and the South Pacific (SP) both exhibit a significant increasing LMI STD trend, with both basins contributing approximately equally to the SH trend. The largest increasing trend among any of the individual TC basins is found over the SP (0.42 kt yr^−1^).

To verify the robustness and reliability of the aforementioned trends in TC LMI variability, the interquartile range (IQR) is applied here to provide descriptive statistics for skewed distributions as the LMI PDF. There are significant increasing trends of LMI IQR from 1981 to 2016 for the globe, with a rate of 0.48 kt yr^−1^ that is about twice the LMI STD trend (Fig. 1a). Similar to the LMI STD, both hemispheric LMI IQRs exhibit significant increasing tendencies (Fig. 1b,c), while the LMI IQR over the WP, SI and SP also increases significantly (Extended Fig. 1). We also investigate TC best tracks from the World Meteorological Organization (WMO)-sanctioned forecast agencies instead of the JTWC for the WP, NI, SI and SP to test the sensitivity of the data sources used. Despite having smaller trends compared to IBTrACS-JTWC, there exist significant increasing tendencies of LMI STD and IQR in IBTrACS-WMO between 1981 and 2016 (Extended Fig. 2).

### Relationship to the LMI PDF change

**Figure 2:**
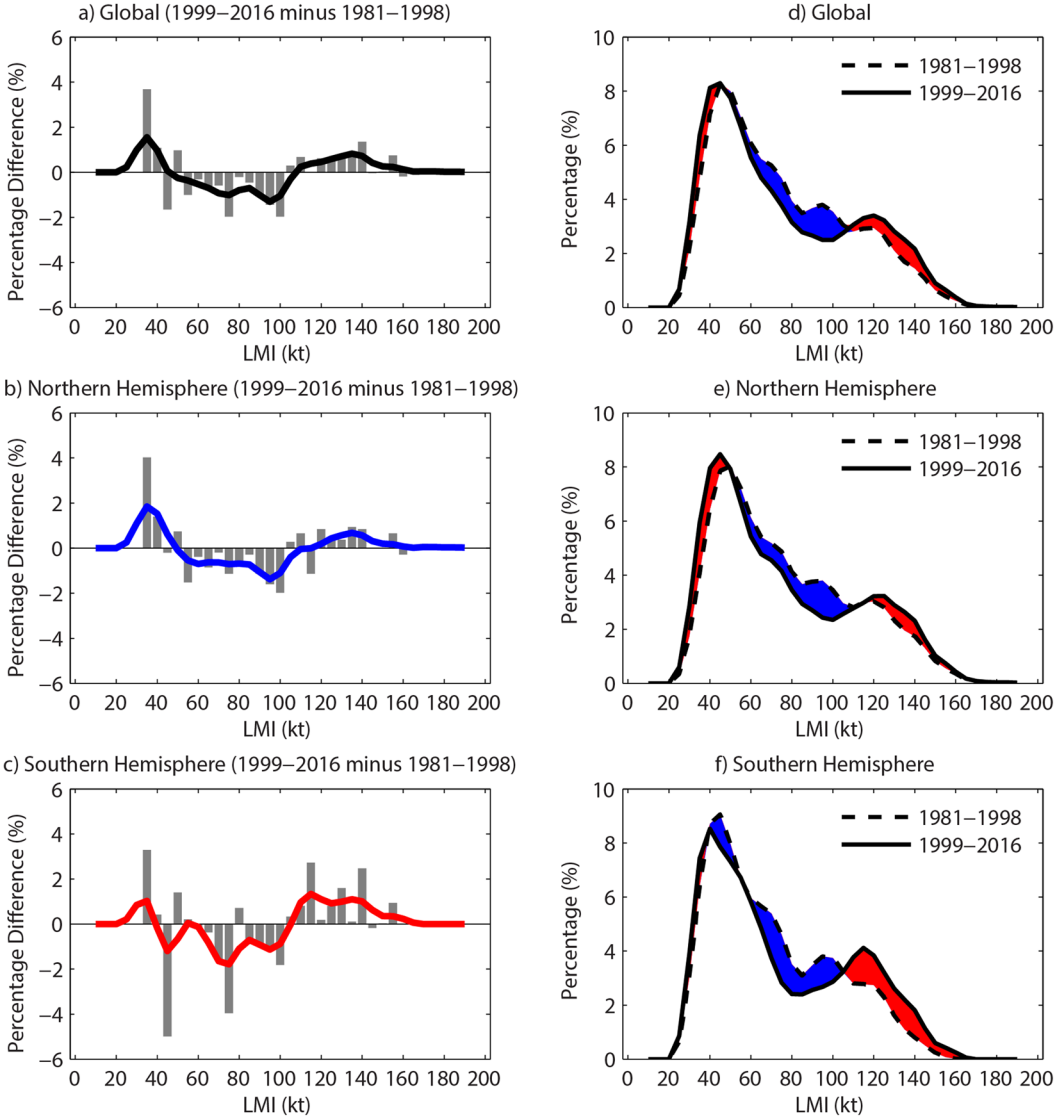
TC LMI distributions and their changes. Grey bars in (**a–c**) represent the raw PDF differences between 1981–1998 and 1999–2016 in 5-kt bins, while black, blue and red solid lines refer to the smoothed PDF differences over the globe (**a**), the Northern Hemisphere (**b**) and the Southern Hemisphere (**c**), respectively. The smoothed lines are obtained by a 5-point low-pass Gaussian filter. The dashed and solid lines in (**d–f**) refer to LMI PDFs in 1981–1998 and 1999–2016 over the globe (**d**), the Northern Hemisphere (**e**) and the Southern Hemisphere (**f**). The positive and negative LMI PDF changes are shown by red and blue areas in (**d–f**), respectively.

The STD of LMI is very sensitive to its PDF, with a flatter (steeper) distribution corresponding to a larger (smaller) STD. The increasing LMI STD is primarily caused by the LMI PDF becoming flatter, which is shown as the LMI PDF difference between 1981–1998 and 1999–2016 (Fig. 2). The smoothed global distribution of the LMI PDF change exhibits a bimodal pattern, with two maxima at 35 kt and 135 kt and a minimum at 95 kt (Fig. 2a). This means that the weakest and strongest TCs around the globe have occurred more frequently in 1999–2016 than in 1981–1998. There has also been a relative reduction in the proportion of moderate TCs around the globe. Note that numerous previous studies have indicated an increasing proportion of strong TCs (e.g. ref.^36^), whereas there is great uncertainty in the trends related to weak TCs in climate projections. Bimodal patterns are also seen in the LMI PDF changes over the NH and SH, although the peaks of the distributions are somewhat different between the hemispheres (Fig. 2b,c). Moreover, the smoothed basin distributions of LMI PDF changes are bimodal in most basins except in the SI which exhibits a decreasing proportion of the weakest TCs (Extended Fig. 3), despite some regional differences in the details. Generally speaking, positive and negative LMI PDF differences can be separated at around 50 kt and 100 kt, indicating that weak tropical storms and category 3–5 TCs are occurring relatively more frequently than strong tropical storms and category 1–2 TCs. Hereafter, weak and strong TCs refer to storms with LMI lower than 50 kt and greater than 100 kt, respectively, while TCs between 50–100 kt are defined as moderate TCs. Note that the bimodal feature in LMI PDF changes was also reported in the comparison of the numbers of simulated NA storm occurrences between control and warmed climates^28^ and the probability density difference of modelled EP and WP storm intensities between different emission scenarios^37^. The observed bimodality of the LMI PDF change is consistent with what would be expected from the TC response to anthropogenic warming.

### Relationship with RI and non-RI TCs

**Figure 3:**
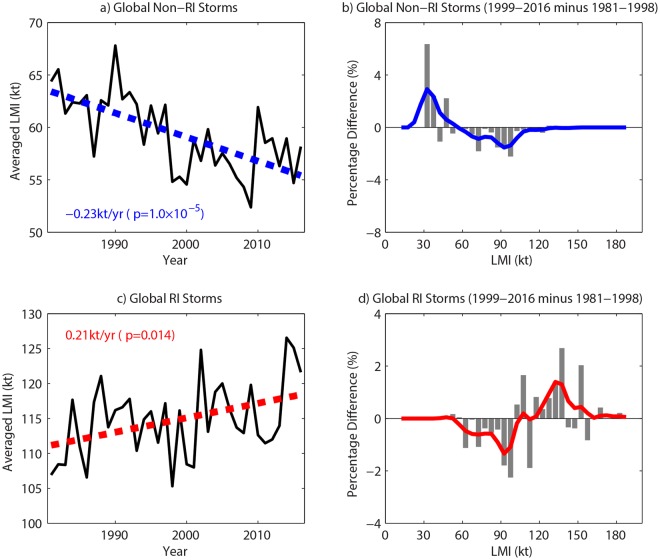
Annual averaged LMI variations and LMI PDF changes for RI and non-RI TCs. (**a**,**b**) and (**c**,**d**) Refer to RI storms and non-RI storms, respectively. In (**a**) and (**c**), dashed lines indicate linear trends from 1981 to 2016, with the rates and associated significance levels displayed in the corners of the plots. In (**b,d**), 5 kt-binned gray bars are raw LMI PDF differences between 1981–1998 and 1999–2016, while solid lines are smoothed by a 5-point low-pass Gaussian filter.

There are distinct LMI PDFs for RI TCs and non-RI TCs, which constitute a bimodal structure of the LMI PDF for all TCs^13,17^. When the annual LMI averages in the period of 1981–2016 are calculated for different TC groups (Fig. 3a,c), opposite trends are represented in global annual-averaged LMI of RI TCs and non-RI TCs. The averaged LMI of RI storms significantly increases at a rate of 0.21 kt yr^−1^, whereas the mean LMI of non-RI storms exhibits a significant decrease of −0.23 kt yr^−1^. Because RI storms on average are more intense than non-RI storms, the above trends indicate that the strong TCs have become stronger, while the weak TCs have gotten weaker. Furthermore, these trends are consistent with the LMI PDF changes between 1981–1998 and 1999–2016 (Fig. 3b,d). The LMI PDF of non-RI storms migrates to lower intensities, with the largest positive and negative changes at 35 kt and 95 kt, respectively. In contrast, the LMI PDF of RI storms skews to higher intensities, resulting in the largest positive and negative differences occurring at 135 kt and 95 kt, respectively. These unimodal patterns of LMI PDF changes for RI TCs and non-RI TCs exhibit consistent signals for individual basins, with the exception of the SI (Extended Fig. 4). The different change in LMI PDF over the SI may be attributed to the change of the satellite viewing angle in 1998, when the Meteosat-5 was repositioned over the Indian Ocean^7^. The unimodal distribution change for RI storms and non-RI storms constitutes the bimodal pattern of the LMI PDF change for all TCs.

## Discussion

The increasing variability of TC LMI, which is displayed as the upward trend in the annual STD of TC LMI, is mainly linked to changes in the TC LMI PDF. The greater proportion of the weakest and strongest TCs to the full TC distribution makes the LMI distribution flatter (Fig. 2d–f), inducing the increasing uncertainty of storm LMI in a warming climate. This bimodal change in the LMI PDF is not only a global phenomenon but also represented in almost all TC basins, which is consistent with several climate modeling results over individual TC basins^28,37^. Our findings are in disagreement with several studies that have investigated the effect of anthropogenic warming on global TC activity through numerical simulations and reported a migration of TC LMI as a whole to higher intensities^18,20–22,24–26^. The primary reason for the disagreement likely results from the detection and tracking methods used for identifying simulated storms in climate models^29^. It is very common to apply a relative humidity, sea level pressure or relative vorticity threshold to distinguish storms from cyclonic perturbations in model outputs^18,20–22,24–26^. These thresholds are dependent on the model resolution, with low thresholds applied to track TCs in low-resolution models^29,38^. However, the systems being identified may not be TCs if too low of a threshold is used. To reduce the uncertainty in identifying modeled weak TCs, these studies did not focus on the projections related to weak TCs. Moreover, as mentioned by refs^29,30^, the higher sensitivity of tracking thresholds for weaker TCs is reduced as the model resolution increases. As a benefit of these high-resolution models, the identifying thresholds used in the models are similar to observations, which can eliminate the uncertainty related to threshold values.

Our study further related the bimodal pattern of the LMI PDF change to the different trends in the annual averaged LMI of RI and non-RI storms. RI (non-RI) storms, which usually exhibit stronger (weaker) intensities, have become increasingly stronger (weaker) during the past two decades. These observed trends are in agreement with ref.^39^, which found, through investigation of the work output of the atmospheric heat engine, that global warming would not induce an overall increasingly stormy atmosphere. It was anticipated that strong storms would become stronger, but weak storms would become weaker. This feature was related to changes in upward motions of different intensities, which showed that in a warming scenario, air masses that could reach the top of the atmosphere were enhanced, whereas those that could not were reduced^39^.

Another mechanism associated with intensity changes of relatively strong and weak TCs is linked to the poleward migration of storm activity^40–42^. In general, TCs generated at lower latitudes can reach greater peak intensities due to a longer time spent in a conducive environment (e.g., warm sea surface temperatures, low vertical wind shear, high mid-level moisture), whereas TCs forming at higher latitudes typically do not achieve as high intensities due to reduced sea surface temperature and enhanced vertical wind shear. Here we take the WP which exhibits the most significant poleward shift of storm tracks and examine trends in the annual averages of WP TC genesis location for RI and non-RI storms (Extended Fig. 5). The significant poleward migration in genesis latitudes of non-RI storms is likely responsible for their significant decreasing intensities. By contrast, there is no significant trend in the latitudinal location for RI storm formation. The strengthening of RI storms is possibly linked to more favorable conditions for tropical development in response to global warming. In other words, the formation position of weak TCs has migrated to higher latitudes, whereas there has not been a latitudinal shift in where strong TCs form. These findings are consistent with weak storms dominating the poleward migration of LMI over the WP^43^.

In addition to physical changes, the trend in weak TC proportion can be induced by temporal inhomogeneities in the best-track data. The evolution of TC observing platforms and improved tracking methodologies may allow for increased observations of weak TCs.

What causes the different LMI trends of RI and non-RI TCs in individual ocean basins remains an open question. Instead of attempting to answer this question for each individual basin, we highlight the non-uniformity of TC LMI PDF changes for anthropogenic warming. These changes further induce increasing variability in TC LMI, which consequently shows an increasingly uncertain behavior in TC intensity. Our results illustrate an increasing proportion of weak TCs, which can provide observations to verify the simulated activity of weak TCs in climate models and to improve tracking algorithms on identifying weak TCs. The simulation of weak TC activity as well as the reanalysis of temporal homogeneous best tracks is an important research issue when it comes to improved understanding of the future impacts of anthropogenic warming on TC frequency and intensity.

## Methods

### Data

TC best-track data are available at https://www.ncdc.noaa.gov/ibtracs/. In order to have one-minute sustained wind estimates for all TC basins, we primarily consider data provided by the National Hurricane Center (NHC)^31^ and the Joint Typhoon Warming Center (JTWC)^32^ (hereafter, IBTrACS-JTWC). For comparison, we also utilize data from the World Meteorological Organization (WMO)-sanctioned forecast agencies for basins outside of the North Atlantic and the eastern North Pacific, which are listed as IBTrACS-WMO.

### LMI

The LMI is defined as the peak one-minute maximum sustained wind achieved by a TC during its lifetime. Only storms with an LMI greater than 34 kt are considered in our study in order to minimize possible influences of the temporal evolution of observational technologies for tropical depressions^44,45^. The percentages of global TCs with a duration less than 24 h and 48 h are 5.7% and 12.4% during 1981–2016, respectively. Our results are not significantly impacted when excluding TCs with a lifetime shorter than 24 h and 48 h, respectively.

### Trends

The trends of time series are obtained using linear least-squares. Two-tailed Student *t*-tests are applied to calculate significance levels. Total global TC numbers average ~85 TCs per year, while those for the Northern Hemisphere and South Hemisphere average around 60 and 25 TCs, respectively.

### LMI PDF and its change

The LMI PDF is defined as the percentage distribution of storm LMI in the nearest 5-kt bins. The LMI PDF change refers to the difference between the LMI PDFs in 1981–1998 and 1999–2016.

## Electronic supplementary material

Supplementary Information
