# Supplementary material for: The increasing variability of tropical cyclone lifetime maximum intensity
Source: Sci Rep. 2018 Nov 9;8:16641. doi: 10.1038/s41598-018-35131-x (PMC6226437; doi:10.1038/s41598-018-35131-x)
Supplement: Supplementary file 1 — Supplementary Information [file 41598_2018_35131_MOESM1_ESM.docx]

**Supplementary Information**

**The increasing variability of tropical cyclone lifetime maximum intensity**

Jinjie Song*^1,2,3^, Philip J. Klotzbach^4^, Jianping Tang^2^, and Yuan Wang^1,2^

^1^ Key Laboratory of Mesoscale Severe Weather, Ministry of Education, China

^2^ School of Atmospheric Sciences, Nanjing University, China

^3^ Joint Center for Atmospheric Radar Research of CMA/NJU, China

^4^ Department of Atmospheric Science, Colorado State University, USA

*Corresponding author: Jinjie Song

Address: 163 Xianlin Road, Nanjing, China, 210023

Email: [songjinjie@nju.edu.cn](mailto:songjinjie@nju.edu.cn)


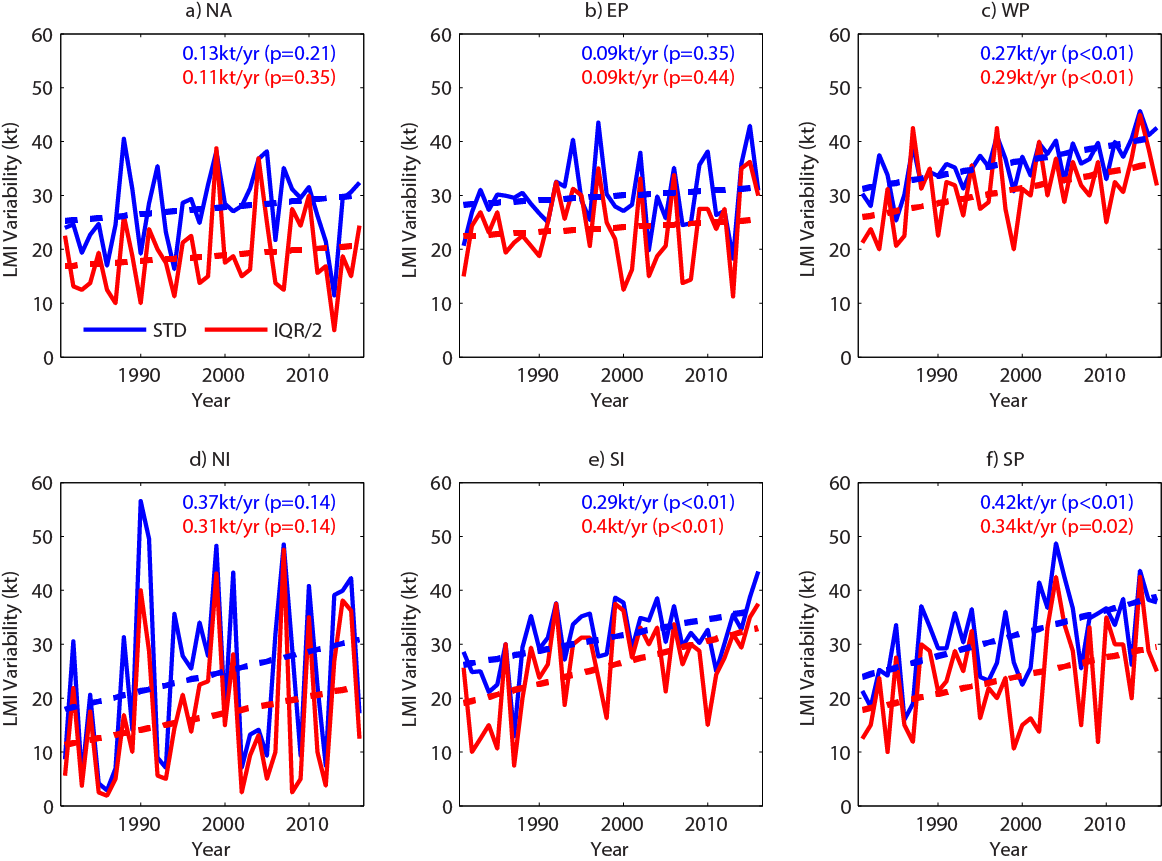


**Extended Figure 1. Time series of annual LMI variability for individual TC basins.** The TC basins displayed are the North Atlantic (a), the eastern North Pacific (b), the western North Pacific (c), the North Indian Ocean (d), the South Indian Ocean (e) and the South Pacific (f). Blue and red lines refer to STD and half IQR, respectively. The slope of the trend line and its significance level are shown in the plots.


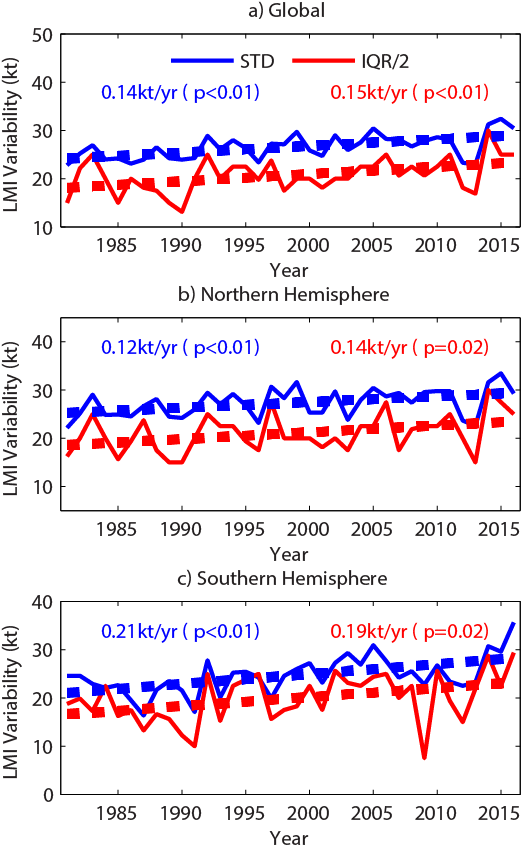


**Extended Figure 2. Increasing variability in TC LMI from the IBTrACS-WMO dataset.** Time series of annual STD and half IQR of TC LMI from 1981 to 2016 and associated linear trend lines over the globe (a), the Northern Hemisphere (b) and the Southern Hemisphere (c). Blue and red lines refer to STD and half IQR, respectively. The slope of the trend line and its significance level are shown in the plots.


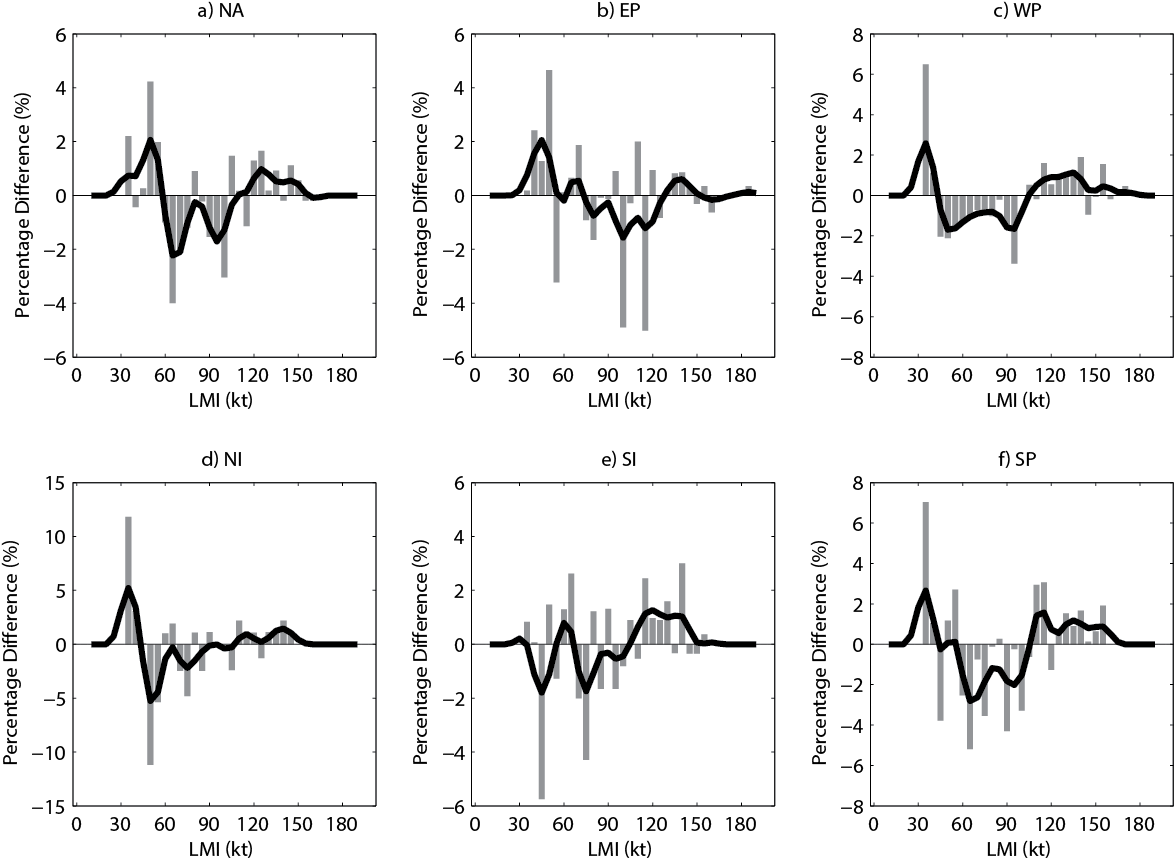


**Extended Figure 3. Changes of regional TC LMI distributions.** Grey bars represent the raw PDF differences between 1981-1998 and 1999-2016 in 5-kt bins, while black lines refer to the smoothed PDF differences obtained by a 5-point low-pass Gaussian filter. The regions displayed are the North Atlantic (a), the eastern North Pacific (b), the western North Pacific (c), the North Indian Ocean (d), the South Indian Ocean (e) and the South Pacific (f).


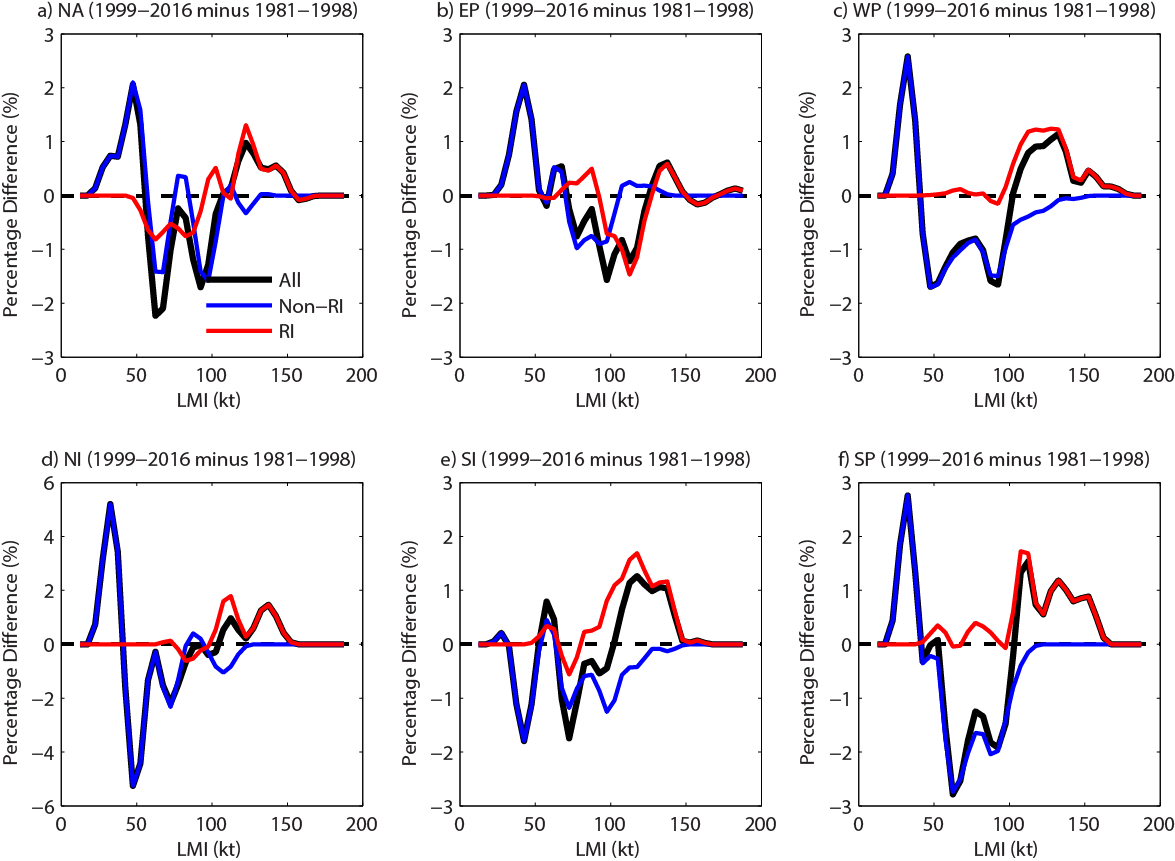


**Extended Figure 4. Changes in smoothed regional LMI distributions for different TC categories.** The black, blue and red lines refer to LMI PDF differences for all, RI and non-RI storms, respectively. The smoothed lines are obtained by a 5-point low-pass Gaussian filter on raw 5 kt-binned data. The TC basins displayed are the North Atlantic (a), the eastern North Pacific (b), the western North Pacific (c), the North Indian Ocean (d), the South Indian Ocean (e) and the South Pacific (f).


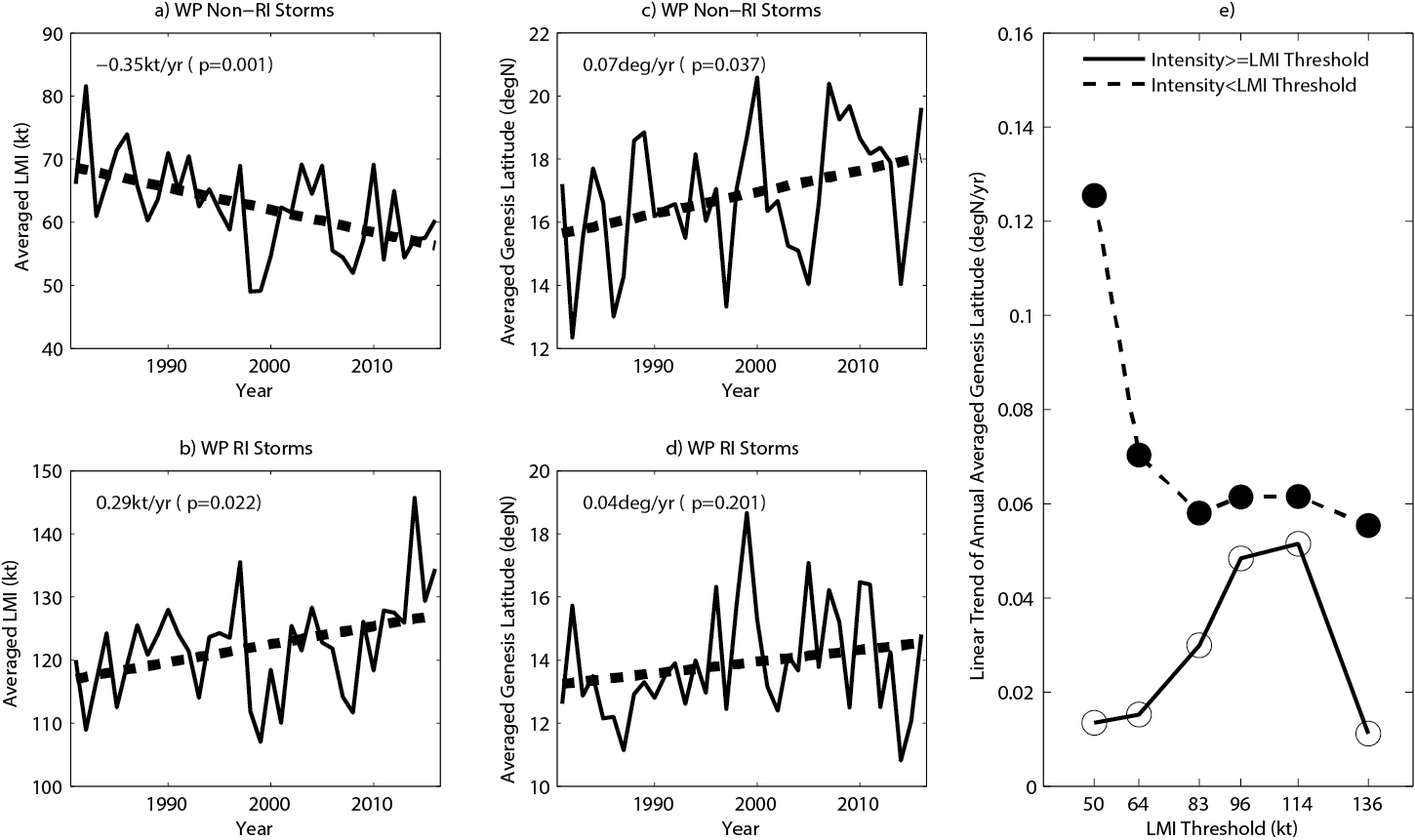


**Extended Figure 5. Annual variation in WP RI and non-RI TCs.** The average LMI and genesis latitude during 1981-2016 are plotted in (a, b) and (c, d), respectively. The dashed lines in (a-d) refer to the linear trend, with the slope and its significance level shown in the upper left corners of the plots. In (e), linear trends in different intensity categories are shown, in which the solid (dashed) line is for TCs with an intensity no less (lower) than the corresponding LMI threshold. The filled dots indicate that the trends are significant at the 0.05 level.
